# The Impact of Hormone Replacement Therapy on Endometrial Pathology: A Retrospective Observational Study

**DOI:** 10.7759/cureus.98415

**Published:** 2025-12-03

**Authors:** Hiba Al Azeez, Mena Abdalla

**Affiliations:** 1 Obstetrics and Gynaecology, Princess Royal University Hospital, London, GBR; 2 Medical Education, Queen's University Belfast, Belfast, GBR; 3 Obstetrics and Gynaecology, Kings College Hospital NHS Foundation Trust, London, GBR

**Keywords:** diagnostic hysteroscopy, endometrial pathology, endometrial thickness (et), hormone replacement therapy (hrt), postmenopausal bleeding

## Abstract

Background and objective

Hormone replacement therapy (HRT) is widely used for managing menopausal symptoms; however, its impact on the endometrium continues to be a clinically significant concern. This study aimed to investigate the association between HRT use and endometrial pathology in women presenting with various gynaecological symptoms.

Methods

A retrospective analysis was conducted involving a cohort of 62 patients who underwent hysteroscopy at the Princess Royal University Hospital. Data on patient demographics, clinical presentation, HRT use, and histological findings were collected and analyzed. Statistical analysis, including chi-square tests and Mann-Whitney U tests, was performed using Python with SPSS-style methodology to assess the relationships between variables.

Results

The mean age of the cohort was 52.7 ± 9.3 years. Of the 62 patients, 13 (21.0%) were on HRT, 11 (17.7%) were not on HRT, and 38 (61.3%) had an unknown HRT status. A statistically significant association was found between HRT status and histological outcomes (χ² = 22.0985, p = 0.0364). Significant associations were also observed between age group and HRT use (χ² = 14.6001, p = 0.0236) and between clinical presentation and HRT use (χ² = 18.1885, p = 0.0058). No significant difference was found in age (p = 0.2340) or endometrial thickness (p = 0.8776) between HRT users and non-users.

Conclusion

This study demonstrates a significant association between HRT use and endometrial pathology. The findings highlight the importance of endometrial surveillance in women on HRT. Further prospective studies with larger sample sizes are needed to validate these findings and to elucidate the long-term effects of different HRT regimens on the endometrium.

## Introduction

Hormone replacement therapy (HRT) is a cornerstone in the management of menopausal symptoms, providing significant relief from vasomotor symptoms and offering protection against osteoporosis [1]. While the therapeutic benefits of HRT are well-established, the relationship between exogenous hormone administration and endometrial health remains an important area of clinical concern and active investigation. The endometrium demonstrates particular sensitivity to hormonal influences, and the use of HRT, especially estrogen-only therapy, has been associated with an increased risk of endometrial hyperplasia and carcinoma [2]. This risk has led to the widespread adoption of combined HRT regimens, wherein progestogen is added to estrogen therapy to counteract the proliferative effects of estrogen on the endometrium [3]. A recent study by Glynne et al., involving 235 postmenopausal women, reported no instances of endometrial hyperplasia or cancer among those using transdermal estradiol combined with micronized progesterone, indicating that appropriate progestogen use may effectively mitigate endometrial risks [4].

Despite the protective effect of progestogens, unscheduled vaginal bleeding remains a common adverse effect in women receiving HRT, often necessitating further investigations to exclude endometrial pathology [5]. Such bleeding can affect up to 40% of HRT users, representing a substantial clinical concern that warrants careful assessment [6]. Hysteroscopy with endometrial biopsy has emerged as the gold standard for evaluating the uterine cavity and obtaining tissue for histological examination, with reported diagnostic accuracy rates exceeding 90% for endometrial cancer detection [7]. Recent meta-analyses have underscored its clinical relevance, showing that in symptomatic postmenopausal women not on HRT, an endometrial thickness threshold of ≤4 mm can effectively exclude endometrial cancer [8]. However, the relationship between HRT use, endometrial thickness, and histological findings requires further investigation, particularly across diverse patient populations [9].

This study aimed to evaluate the relationship between HRT use and endometrial pathology in a cohort of women presenting with various gynecological symptoms at a single institution. We hypothesized that there would be measurable differences in the histological findings between women receiving HRT and those not receiving HRT, and that these differences may have important clinical implications for patient management and surveillance protocols.

## Materials and methods

Study design and population

**Figure 1:**
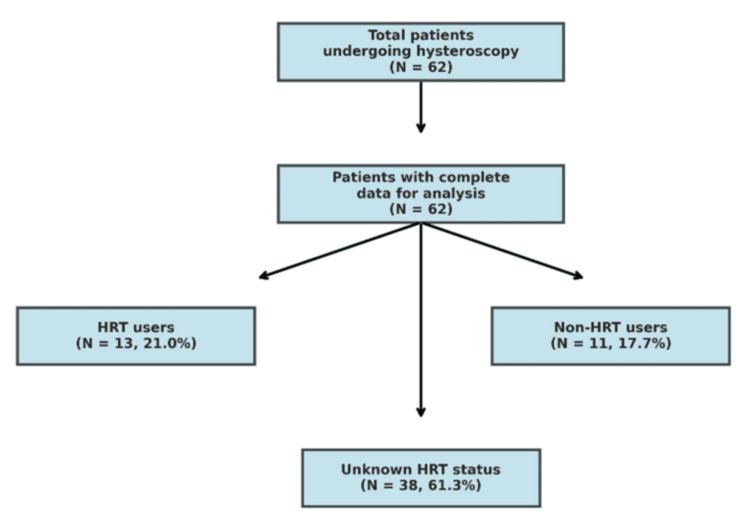
Study flow diagram The flow diagram depicts the selection and categorization of 62 patients who underwent hysteroscopy at Princess Royal University Hospital. Patients were categorized by HRT status with frequencies and percentages: 13 (21.0%) receiving HRT, 11 (17.7%) not receiving HRT, and 38 (61.3%) with unknown HRT status. Data presented as n (%). Statistical significance for associations was set at p<0.05 HRT: hormone replacement therapy

A retrospective observational study was conducted at Princess Royal University Hospital, NHS Foundation Trust. The study population comprised all women who underwent hysteroscopy for gynecological symptoms and had complete data recorded in the electronic patient record system. A total of 62 patients were included in the final analysis after applying the inclusion and exclusion criteria. The study flow diagram (Figure 1) illustrates the patient selection process and categorization by HRT status.

Data collection

Data were retrospectively collected from electronic patient records using a standardized data collection form. The collected variables included patient demographics (age, BMI, parity), clinical presentation (such as post-menopausal bleeding, heavy menstrual bleeding, irregular bleeding), detailed HRT information, transvaginal ultrasound findings including endometrial thickness measurements, hysteroscopic findings, and histological results of endometrial biopsies.

HRT status was categorized into three groups: "On HRT" (patients currently using any form of hormone replacement therapy), "No HRT" (patients explicitly documented as not using HRT), and "Unknown" (patients with insufficient documentation regarding HRT use). Clinical presentations were categorized into post-menopausal bleeding, heavy menstrual bleeding, irregular bleeding, and other presentations.

Statistical analysis

Data were analyzed using SPSS Statistics (IBM Corp., Armonk, NY). Descriptive statistics were used to summarize the baseline characteristics of the study population, with continuous variables presented as mean ± standard deviation (SD) and categorical variables as frequencies (n) and percentages (%). Categorical variables were compared using the chi-square test, with Fisher's exact test applied when expected cell counts were fewer than five. Continuous variables were compared using the Mann-Whitney U test due to the non-normal distribution of the data. Spearman correlation analysis was performed to assess the relationship between age and endometrial thickness. A p-value of <0.05 was considered statistically significant for all analyses.

## Results

Patient characteristics

A total of 62 patients were included in the study. The demographic and clinical characteristics of the study population are presented in Table 1. The mean age of the cohort was 52.7 ± 9.3 years (range: 24-75 years). The median age was 54.0 years with an interquartile range (IQR) of 47.0-59.0 years. Regarding age distribution, 14 patients (22.6%) were younger than 45 years, 23 (37.1%) were between 45 and 54 years, 21 (33.9%) were between 55 and 64 years, and three patients (4.8%) were 65 years or older.

**Table 1:** Demographic and clinical characteristics of the study population (N = 62) Statistical significance set at p<0.05 SD: standard deviation; IQR: interquartile range; BMI: body mass index; HRT: hormone replacement therapy

Characteristic	Values
Age, years	
Mean ± SD	52.7 ± 9.3
Median (IQR)	54.0 (47.0-59.0)
Range	24-75
Age groups, years, n (%)	
<45	14 (22.6)
45-54	23 (37.1)
55-64	21 (33.9)
≥65	3 (4.8)
BMI, kg/m²	
Available data, n (%)	1 (1.6)
Parity	
Available data, n (%)	27 (43.5)
Median (IQR)	2.0 (1.0-3.0)
Endometrial thickness, mm	
Available data, n (%)	41 (66.1)
Mean ± SD	7.4 ± 3.9
Median (IQR)	6.6 (4.6-9.4)
Range	2.8-18.0
HRT status, n (%)	
On HRT	13 (21.0)
No HRT	11 (17.7)
Unknown/not documented	38 (61.3)
Clinical presentation, n (%)	
Post-menopausal bleeding	10 (16.1)
Heavy menstrual bleeding	4 (6.5)
Irregular bleeding	7 (11.3)
Other	41 (66.1)
Histological findings, n (%)	
Normal	22 (35.5)
Other	20 (32.3)
Unknown	11 (17.7)
Inadequate sample	5 (8.1)
Proliferative	2 (3.2)
Secretory	1 (1.6)
Atrophic	1 (1.6)

Regarding HRT status, 13 patients (21.0%) were documented as current HRT users, 11 patients (17.7%) were confirmed as not using HRT, and 38 patients (61.3%) had unknown or inadequately documented HRT status. The most common clinical presentation was categorized as "Other" (n = 41, 66.1%), followed by post-menopausal bleeding (n = 10, 16.1%), irregular bleeding (n = 7, 11.3%), and heavy menstrual bleeding (n = 4, 6.5%).

Endometrial thickness measurements were available for 41 patients (66.1% of the cohort). The mean endometrial thickness measured 7.4 ± 3.9 mm, with a median of 6.6 mm and an IQR of 4.6-9.4 mm. Values ranged from 2.8 mm to 18.0 mm, indicating considerable variation in endometrial thickness across the study population.

Results of statistical analysis

**Figure 2:**
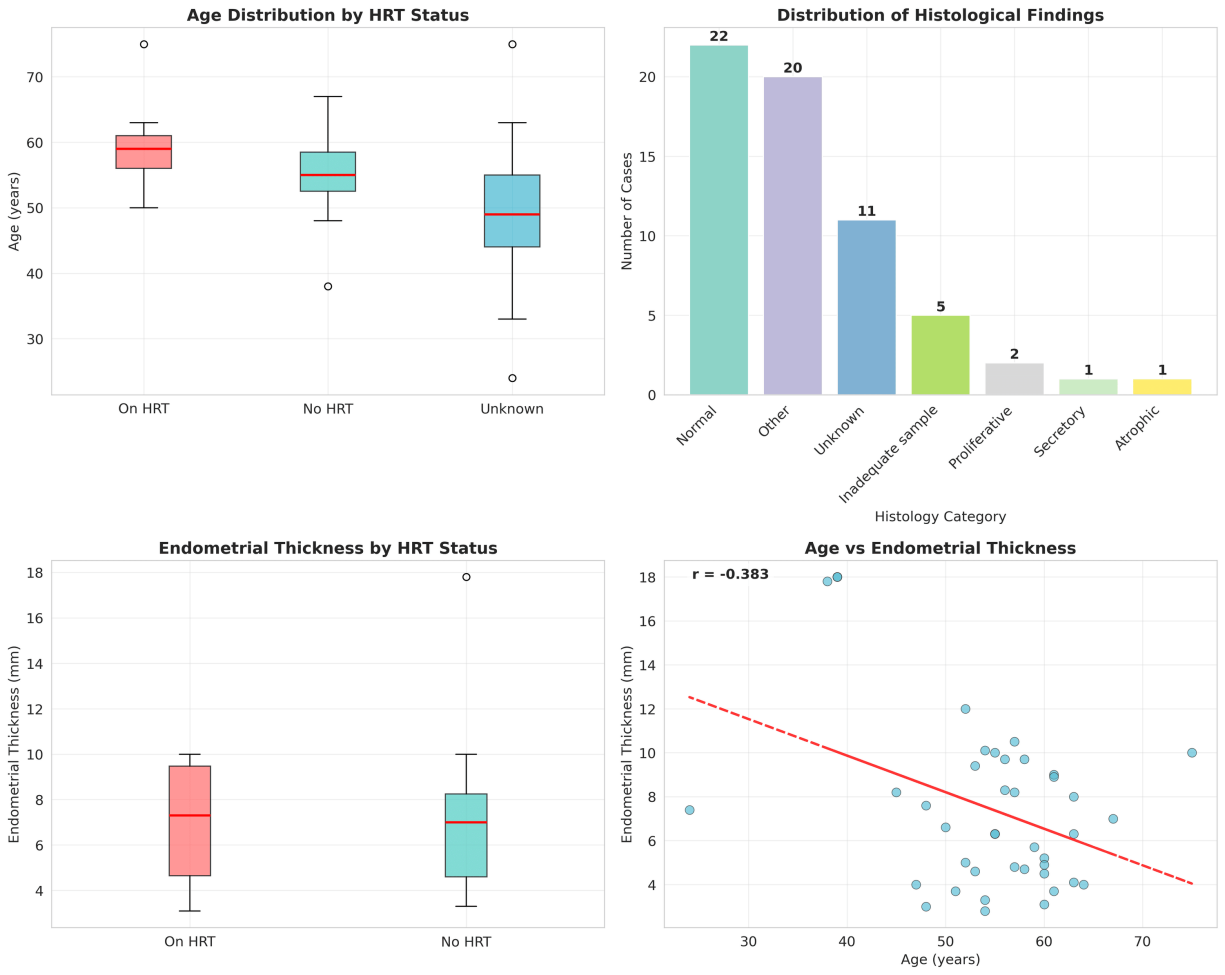
Statistical analysis plots (A) Age distribution by HRT status presented as box plots with median values: HRT users: 59.0 years, non-users: 55.0 years, unknown status: 49.0 years (Mann-Whitney U test, p = 0.2340). (B) Distribution of histological categories, with normal endometrium being the most common (n = 22, 35.5%). (C) Endometrial thickness comparison between HRT users (median: 7.3 mm) and non-users (median: 7.0 mm), showing no significant difference (Mann-Whitney U test, p = 0.8776). (D) Scatter plot of age versus endometrial thickness, with regression line showing weak negative correlation (Spearman ρ = -0.179, p = 0.2628). Data presented as median values for continuous variables and frequencies (n) with percentages (%) for categorical variables. Statistical significance set at p<0.05 HRT: hormone replacement therapy

The comprehensive statistical analysis results are summarized in Figure 2, which presents a four-panel visualization of the key findings, and Table 2, which provides detailed statistical outcomes.

**Table 2:** Summary of statistical results Chi-square tests performed for categorical variables: Mann-Whitney U tests for continuous variables, and Spearman correlation for correlation analysis. Statistical significance set at p<0.05 HRT: hormone replacement therapy

Statistical test	Variables compared	Test statistic	P-value	Statistical significance (p<0.05)	Clinical interpretation
Chi-square test	HRT status vs. histology category	χ² = 22.10	0.0364	Yes	Significant association between HRT use and endometrial histology
Chi-square test	Age group vs. HRT status	χ² = 14.60	0.0236	Yes	Significant association between age and HRT use
Chi-square test	Clinical presentation vs. HRT status	χ² = 18.19	0.0058	Yes	Significant association between clinical presentation and HRT use
Mann-Whitney U test	Age: HRT users vs. non-users	U = 92.5	0.234	No	No significant difference in age between groups
Mann-Whitney U test	Endometrial thickness: HRT users vs. non-users	U = 69.0	0.8776	No	No significant difference in endometrial thickness between groups
Spearman correlation	Age vs. endometrial thickness	ρ = -0.179	0.2628	No	Weak negative correlation, not statistically significant

Association between HRT and histology

**Figure 3:**
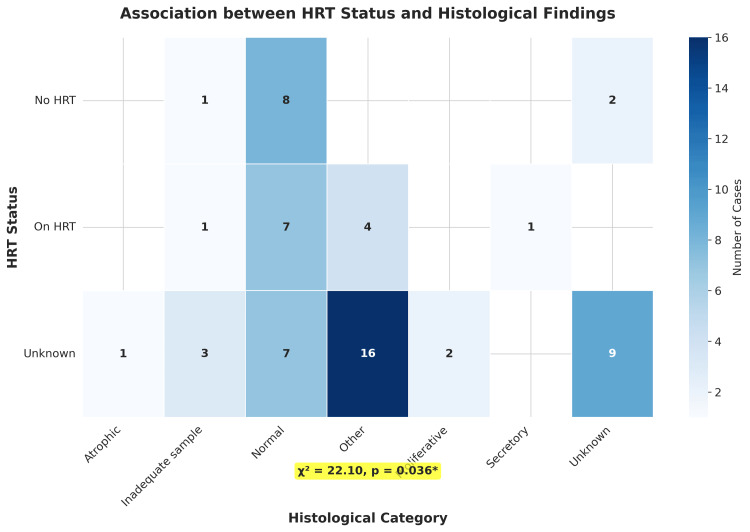
Association between HRT status and histological findings Heatmap displaying the contingency table analysis of HRT status versus endometrial histology categories with frequencies (n) shown in each cell. The analysis revealed a statistically significant association (χ² = 22.10, p = 0.036, p<0.05). Normal endometrium was the predominant finding across all HRT groups, with no cases of endometrial hyperplasia or cancer identified. Color intensity represents the number of cases (n) in each category, with darker blue indicating higher frequencies. Data presented as frequencies (n) with statistical significance set at p<0.05 HRT: hormone replacement therapy

A statistically significant association was identified between HRT status and the histological category of the endometrial biopsy (χ² = 22.0985, degrees of freedom = 12, p = 0.0364). The distribution of histological findings among the different HRT groups is illustrated in Figure 3. Among patients receiving HRT, the most common histological finding was "Normal" endometrium (n = 7, 53.8%), followed by "Other" findings (n = 4, 30.8%). In the no-HRT group, "Normal" endometrium was found in eight patients (72.7%), with "Other" findings in two patients (18.2%).

The histological categories across the entire cohort demonstrated that normal endometrium was the most frequent finding (n = 22, 35.5%), followed by "Other" findings (n = 20, 32.3%), "Unknown" results (n = 11, 17.7%), "Inadequate sample" (n = 5, 8.1%), "Proliferative" endometrium (n = 2, 3.2%), "Secretory" endometrium (n = 1, 1.6%), and "Atrophic" endometrium (n = 1, 1.6%).

Association between age and clinical presentation

There was a statistically significant association between age group and HRT status (χ² = 14.6001, degrees of freedom = 6, p = 0.0236). The analysis revealed that HRT use was most prevalent in the 55-64 age group, where nine out of 21 patients (42.9%) were receiving HRT. In contrast, no patients younger than 45 years were documented as receiving HRT, which is consistent with the typical age of menopause onset.

Clinical presentation was also significantly associated with HRT status (χ² = 18.1885, degrees of freedom = 6, p = 0.0058). Post-menopausal bleeding was observed in five patients (45.5%) in the no-HRT group and four patients (30.8%) in the HRT group. This finding suggests that post-menopausal bleeding occurs in both HRT users and non-users, emphasizing the importance of investigation regardless of HRT status.

Comparison​​​​ of continuous variables

Despite the significant associations found with categorical variables, there was no statistically significant difference in the median age between HRT users (59.0 years) and non-users (55.0 years) using the Mann-Whitney U test (U = 92.5, p = 0.2340). This finding suggests that while age groups demonstrate different patterns of HRT use, the actual age distributions between users and non-users overlap considerably.

Similarly, there was no significant difference in the median endometrial thickness between HRT users (7.3 mm) and non-users (7.0 mm) (U = 69.0, p = 0.8776). This finding is particularly noteworthy as it suggests that HRT use, at least in this cohort, does not significantly alter endometrial thickness measurements as detected by transvaginal ultrasound.

Correlation analysis

Spearman correlation analysis between age and endometrial thickness revealed a weak negative correlation (ρ = -0.179, p = 0.2628) that was not statistically significant. This suggests that in this cohort, age and endometrial thickness are not strongly related, which may reflect the complex interplay of hormonal, metabolic, and individual factors affecting endometrial characteristics.

## Discussion

This study demonstrates a significant association between HRT use and endometrial histology in a cohort of women undergoing hysteroscopy for various gynecological symptoms. This finding aligns with the established understanding of the effects of exogenous hormones on the endometrium and reinforces the importance of ongoing endometrial surveillance in women receiving HRT [10]. The significant association between age and HRT use observed in our study is consistent with clinical expectations, as HRT is typically initiated around the time of menopause. The finding that HRT use was most common in the 55-64 age group reflects the typical demographic of women seeking hormone therapy for menopausal symptoms. This age-related pattern is important for clinical practice as it identifies the population most likely to require endometrial monitoring [11].

One of the most intriguing findings of our study is the lack of a significant difference in endometrial thickness between HRT users and non-users. This observation contrasts with some previous studies that have suggested HRT may influence endometrial thickness. Recent research by Glynne et al. similarly found that endometrial thickness was not significantly associated with estradiol dose in women using transdermal HRT, supporting our findings [4]. This may suggest that the relationship between HRT and endometrial thickness is more complex than previously understood and may be influenced by factors such as the type of HRT regimen, duration of use, and individual patient characteristics [12]. The absence of endometrial hyperplasia or cancer in our cohort is reassuring and consistent with recent large-scale studies. Glynne et al. reported no cases of endometrial hyperplasia or cancer in 235 women with unscheduled bleeding on transdermal estradiol plus micronized progesterone [4]. This finding supports the safety profile of appropriately prescribed combined HRT regimens and suggests that the addition of progestogen effectively mitigates the proliferative effects of estrogen on the endometrium [13].

Our study has several important limitations that must be acknowledged. First, the retrospective design limits our ability to establish causal relationships and may introduce selection bias. The relatively small sample size of 62 patients, while adequate for statistical analysis, limits the generalizability of our findings to broader populations. Additionally, the large proportion of patients with unknown HRT status (61.3%) represents a significant limitation that may have affected the power of our statistical analyses. The heterogeneity of HRT regimens used by patients in our study is another limitation. We were unable to distinguish between specific HRT types (e.g., estrogen-only vs. combined therapy), routes of administration (oral, transdermal, vaginal), or duration of use. This variability may have obscured important differences in how particular HRT formulations influence endometrial outcomes. The classification of many histology results as “Other” or “Unknown” may have limited our ability to detect more subtle associations between HRT use and specific histological patterns. Future studies would benefit from more standardized histological reporting and classification systems.

Despite these limitations, our study provides valuable insights into the relationship between HRT and endometrial pathology in a real-world clinical setting. The findings underscore the importance of individualized assessment and monitoring of women on HRT, particularly those presenting with unscheduled bleeding. The significant associations we observed between HRT status and both age and clinical presentation provide important clinical context for the management of these patients. The clinical implications of our findings are multifaceted. The significant association between HRT use and endometrial histology reinforces the importance of appropriate patient selection, counseling, and monitoring for women considering or using HRT [14]. Healthcare providers should maintain a high index of suspicion for endometrial pathology in women on HRT who present with unscheduled bleeding, regardless of endometrial thickness measurements.

Future research should prioritize larger, prospective studies with detailed characterization of HRT regimens, including type, dose, duration, and route of administration. Such studies would help to refine our understanding of the relationship between specific HRT formulations and endometrial outcomes. Additionally, longer-term follow-up studies are needed to assess the cumulative effects of HRT on endometrial health and to identify optimal surveillance strategies for different patient populations [15]. The integration of molecular markers and advanced imaging techniques may also provide new insights into the mechanisms underlying HRT effects on the endometrium. Recent advances in endometrial cancer classification and risk stratification may inform future approaches to monitoring women on HRT [9].

## Conclusions

This retrospective observational study demonstrates a statistically significant association between HRT use and endometrial histology in women undergoing hysteroscopy. While no cases of endometrial hyperplasia or cancer were identified, the significant relationships between HRT status, age, and clinical presentation offer valuable clinical insights regarding patient management. The findings emphasize the continued importance of individualized endometrial surveillance in women receiving HRT, particularly those presenting with unscheduled bleeding. Further prospective studies with larger sample sizes and detailed HRT characterization are warranted to confirm these findings and optimize surveillance strategies for women receiving HRT.
